# The utility of upadacitinib to treat refractory hidradenitis suppurativa in an obese patient

**DOI:** 10.1016/j.jdcr.2024.08.002

**Published:** 2024-08-30

**Authors:** Zahidul Islam, Peter Y. Ch’en, Solbie Choi, Yasmine Oprea, Kristina Campton

**Affiliations:** Division of Dermatology, Albert Einstein College of Medicine, Bronx, New York

**Keywords:** anti-TNF therapy, BMI, hidradenitis suppurativa, JAK, Janus kinase (JAK) inhibitors, obesity, refractory, upadacitinib

## Introduction

Hidradenitis suppurativa (HS) is a chronic inflammatory skin condition characterized by recurrent painful nodules, abscesses, and sinus tracts. The precise etiology of HS remains incompletely understood, but it is thought to involve a combination of genetic, immunological, and environmental factors.

Janus kinase (JAK) inhibitors have emerged as promising treatment options for moderate-to-severe cases of treatment-refractory HS. Upadacitinib is a second-generation JAK inhibitor that primarily targets the JAK1 enzyme, blocking downstream signaling of pro-inflammatory cytokines such as interleukin (IL)-6, IL-23, and interferon-gamma.^1^ The off-label use of upadacitinib for treatment-resistant HS has been reported, showing evidence of cutaneous improvement, decreased HS flares, and reduced pain. A clinical trial conducted to assess the efficacy of upadacitinib in moderate-to-severe HS demonstrated that compared to placebo, treatment with upadacitinib led to reductions in abscess and inflammatory nodule count and in the Hidradenitis Suppurativa Physician’s Global Assessment score while being well-tolerated by patients.^2^ Additionally, a retrospective cohort study of 20 patients with moderate-to-severe HS treated with upadacitinib found that 75% of patients achieved Hidradenitis Suppurativa Clinical Response 50 at week 4 of treatment and 100% of patients reached Hidradenitis Suppurativa Clinical Response 50 by week twelve and that a higher dose of 30 mg was associated with improved disease control in Hurley stage III HS.^3^ Thus, upadacitinib may present a promising alternative for patients who have failed therapy with tumor necrosis factor-α inhibitors or IL-17 blockers like secukinumab; however, data are limited. To our current knowledge, no studies have examined the use of higher doses of upadacitnib in patients with HS and obesity. Here, we report a unique case of refractory HS successfully treated with 45 mg of upadacitinib daily.

## Case presentation

A 38-year-old male, current smoker, with a history of obesity (body mass index (BMI): 40.5), type II diabetes, hypertension, hyperlipidemia, acne, pilonidal cyst, and HS for 11 years presented to our clinic with flares in the groin and buttocks. On physical exam, he had tender nodules in the bilateral inguinal folds, and tender, hyperpigmented nodules and sinus tracts on his buttocks. He reported severe pain, especially when sitting down, from these lesions and rated it a 9/10. His international HS Severity Score System (IHS4) was an 11, IL-6 was 22.02 pg/mL, erythrocyte sedimentation rate was 67 mm/h, and C-reactive protein (CRP) was 8.1 mg/dL. He was started on chlorhexidine/clindamycin gel, levofloxacin/rifampin, finasteride, and infliximab. However, he stopped taking dual antibiotics due to experiencing vomiting, diarrhea, and gastrointestinal aches. He also reported respiratory distress and vomiting after infliximab infusions and was switched to double-dose adalimumab. Trimethoprim/sulfamethoxazole, ertapenem, ceftazadime/avibactam, and double-dose adalimumab were all trialed with no clinically satisfactory improvement.

**Fig 1:**
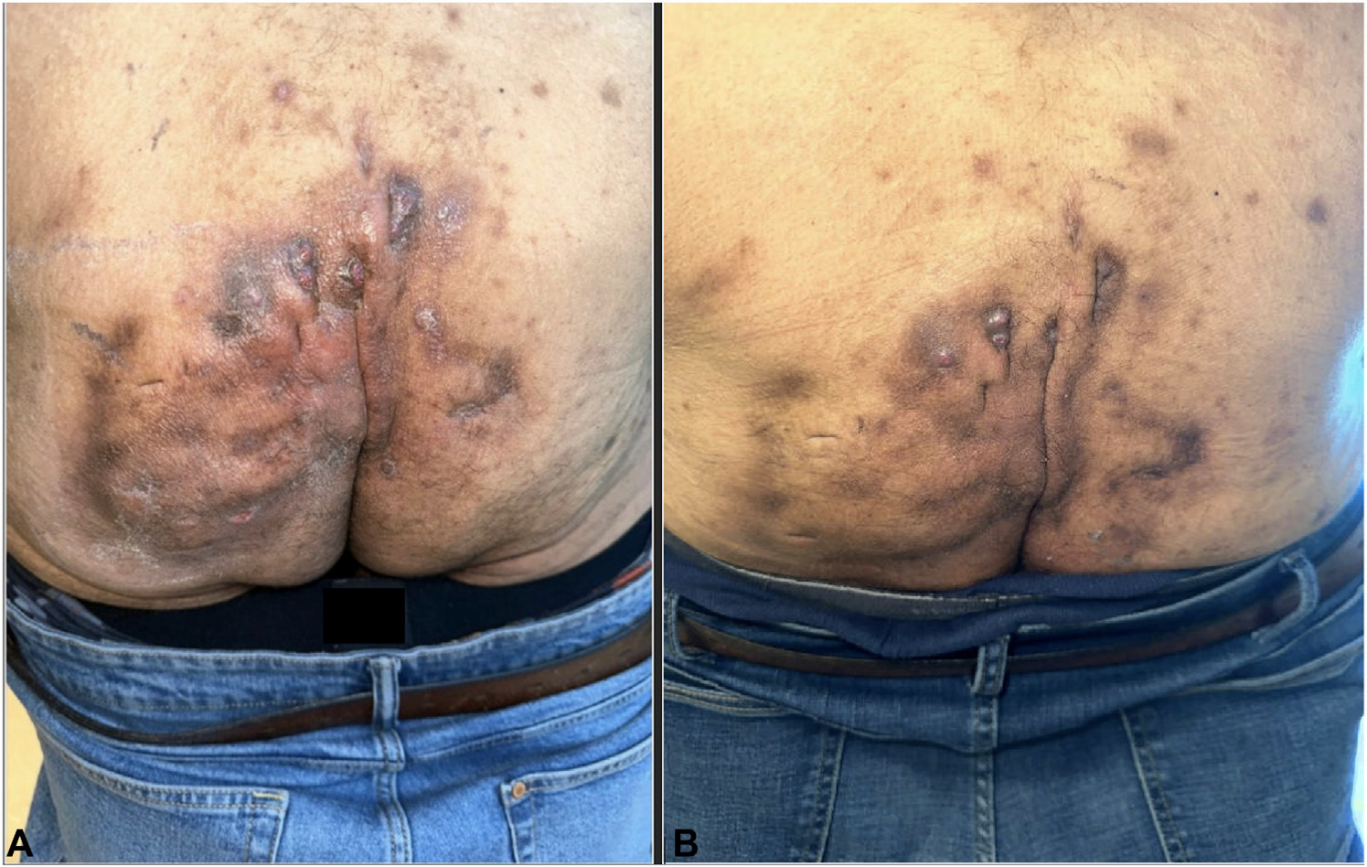
Improvement of inflammation, erythema, and drainage from initial visit (**A**) to 2-month follow-up visit (**B**).

He was lost to follow-up and resumed treatment upon returning to the clinic after 2 years. A week prior, he had visited the emergency room for a severe HS flare and was given doxycycline and oxycodone-acetaminophen. In-clinic, his physical exam was like his initial presentation 2 years ago, now with increased extensiveness of buttocks lesions and scarring, and the addition of inflammatory nodules present on his scalp. We continued antibiotic treatment and added benzoyl peroxide, finasteride, and upadacitinib 45 mg daily to his regimen. Upadacitinib 45 mg was chosen due to the previous failure of double-dose adalimumab, the adverse reaction of infliximab, patient preference, and BMI. At a 2-month follow-up, he reported a considerable decrease in pain, drainage, and frequency of flares and stated, “the best his HS has been in years.” No side effects were reported and his physical exam revealed decreased erythema, tenderness, and active disease in the groin, scalp, and buttocks areas (Fig 1). Only the buttocks area had both pretreatment and post-treatment photos. His IHS4 score at follow-up was a 4, IL-6 level was 6.51 pg/mL, erythrocyte sedimentation rate was 65 mm/h, and CRP was 0.7 mg/dL.

## Discussion

Data on the usage of upadacitinib for HS treatment are limited. Previously, upadacitinib for HS was examined with a maximum dosage of 30 mg at 12-week intervals that showed positive clinical responses in a phase-II trial and retrospective study.^2^^,^^3^ Furthermore, a proteomic analysis found a significant reduction of signaling molecules involved with known inflammatory pathways associated with HS.^4^

Compared to anti-tumor necrosis factor therapies, which require either infusions or injections, the oral formulation of upadacitinib may be more palatable for some patients, especially those averse to needles. One study found adalimumab efficacy to be inversely correlated with BMI, which suggests that the frequency of injections must be increased or requires a switch to weight-based infliximab, both of which were not effective in our patient.^5^ Upadacitinib may be more easily integrated into a patient’s schedule than infliximab, as it can eliminate lengthy trips to infusion centers, infusion reactions, and the development of antidrug antibodies, resulting in a loss of response.^6^ However, upadacitinib should be avoided in patients with a significant cardiac history or severe hepatic impairments.

Our patient received a 45 mg daily dose of upadacitinib, the highest dose used in HS so far in the literature. Although the relationship between BMI and upadacitinib efficacy has not yet been studied, it is possible that a higher dose was necessary due to his high BMI. Reductions in IHS4 from 11 to 4 and decreases in inflammatory lab markers, especially the CRP and IL-6, were observed. Our findings are not only consistent with previous studies, but they also provide a new avenue of exploration in HS therapeutics. We demonstrate the real-world feasibility and effectiveness of 45 mg upadacitinib with minimal side effects. This case pushes the bounds of upadacitinib usage for moderate-to-severe HS, for which patients commonly are on multiple concomitant therapies or have failed previous anti-inflammatory therapies such as adalimumab or infliximab.

Overall, it is important to value the patient’s preferences during shared decision-making. Our paper expands upon the literature as we examine the efficacy of 45 mg upadacitinib for the treatment of refractory HS in an obese patient and provides another treatment option; however, it is limited to a single case. Further trials examining upadacitnib’s dose-response relationship, efficacy-BMI relationship, and comparison against biologics are warranted.

## Conflicts of interest

None disclosed.
